# FoodMicroDB: A microbiome database for composition and time‐series research in food

**DOI:** 10.1002/imo2.40

**Published:** 2024-11-01

**Authors:** Yahui Li, Hujie Lyu, Haifei Yang, Zhicheng Ju, Chuang Ma, Huiyu Hou, Yao Wang, Yuanping Zhou, Yunyun Gao, Junbo Yang, Shanshan Xu, Defeng Bai, Hao Luo, Salsabeel Yousuf, Tianyuan Zhang, Jiani Xun, Meiyin Zeng, Heyuan Qi, Tong Chen, Yong‐Xin Liu

**Affiliations:** ^1^ Agricultural Genomics Institute at Shenzhen Chinese Academy of Agricultural Sciences Shenzhen China; ^2^ Department of Life Sciences Imperial College of London London UK; ^3^ College of Life Sciences Qingdao Agricultural University Qingdao China; ^4^ Department of Ocean Science The Hong Kong University of Science and Technology Hong Kong SAR China; ^5^ Anhui Agricultural University Hefei China; ^6^ Zhanjiang Key Laboratory of Human Microecology and Clinical Translation Research, The Marine Biomedical Research Institute, College of Basic Medicine Guangdong Medical University Zhanjiang Guangdong China; ^7^ School of Food and Biological Engineering Hefei University of Technology Hefei China; ^8^ Institute of Microbiology Chinese Academy of Sciences Beijing China; ^9^ State Key Laboratory for Quality Ensurance and Sustainable Use of Dao‐di Herbs, National Resource Center for Chinese Materia Medica China Academy of Chinese Medical Science Beijing China

**Keywords:** database, food microbiome, fermented food, microbial composition, microbiome analysis, time‐series

## Abstract

Microorganisms are crucial for food fermentation, preservation, and safety, directly impacting human health. The number of studies on the food microbiome has surged recently, along with a substantial increase in data. However, there is a notable lack of databases specialized for this field. To address this gap, we developed Food Microbiome Database (FoodMicroDB), a platform aimed at enhancing the reusability and accessibility of food microbiome data through comprehensive data management and data visualization tools. FoodMicroDB aggregates 6358 amplicon data from 108 meta‐taxonomic projects covering 62 foods, and harbors 4710 taxa of bacteria, archaea, and fungi. The collected data were consistently analyzed and curated, then visualized using versatile utilities, including unique tools for visualizing microbial composition and time‐series microbiome data. It also includes advanced modules for microbial abundance analysis, cross‐host abundance comparison, and cross‐food analysis. FoodMicroDB will be a valuable resource platform for the food microbiome research field. The database is freely accessible at: https://www.bic.ac.cn/FoodMicro/.

## Highlights

- FoodMicroDB is a freely accessible database specialized for food microbiome research, organizing microbiome data by project and food/organism.
- The platform ensures consistent data analysis and metadata curation, facilitating cross‐host comparisons.
- It provides versatile data visualization modules, including unique tools for visualizing composition and time‐series microbiome data.

## Graphical abstract

FoodMicroDB is a freely accessible database specialized for food microbiome research, including 6358 amplicon data from 108 meta‐taxonomic projects covering 62 foods and 4710 taxa of bacteria, archaea, and fungi. The platform ensures consistent data analysis and metadata curation, facilitating cross‐host comparisons. It features versatile data visualization modules, including unique tools for visualizing composition and time‐series microbiome data.

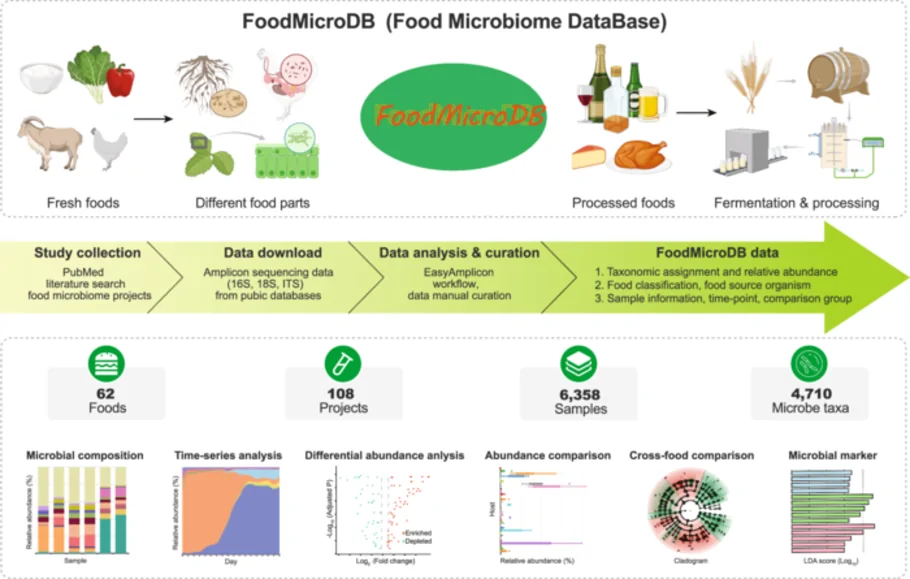

## 1 INTRODUCTION

Food encompasses both raw agricultural products and processed items, such as fermented and packaged foods. The food microbiome, comprising microorganisms associated with various food types, plays a critical role in plant and animal health, food fermentation, and production processes [1]. Advances in targeted sequencing and metagenomics have provided deep insights into microbial ecosystems of foods, elucidating their impact on flavor, preservation, safety, and ultimately human health [2, 3]. For example, the health benefits of fermented food are increasingly recognized [4, 5, 6, 7, 8, 9]. The characterization of microbiota during key fermentation stages is crucial for consistent food production and, therefore, extensively studied [10, 11]. Microbiomes on fruit or vegetable surfaces are extensively studied for postharvest preservation [12, 13], while plant rhizosphere microbiomes are vital for plant growth and flavor [14, 15]. Studies on the livestock digestive tract microbiomes are essential for ensuring the quality and safety of animal‐sourced food [16, 17, 18].

With the growing importance of food system microbiome studies, a significant amount of amplicon and metagenomic sequencing data has been generated. Raw sequencing data are often deposited into public databases, including NCBI Sequence Read Archive (SRA, https://www.ncbi.nlm.nih.gov/sra) [19], European Nucleotide Archive (ENA, https://www.ebi.ac.uk/ena) [20], and CNCB Genome Sequence Archive (GSA, https://ngdc.cncb.ac.cn/gsa/) [21]. Additionally, microbiome databases like MGnify [22] and gcMeta [23] have archived processed data based on the host habitats, while MG‐RAST [24] and Qiita [25] offer additional functional analysis tools. These databases significantly enhancing data accessibility and reusability. However, they are not specifically designed for food microbiome datasets and lack curation of food‐associated metadata. FoodMicroBioNet [26], on the other hand, hosts a comprehensive food meta‐taxonomic data set from 251 published studies. Yet, it is only accessible via an R shiny application, which might not be user‐friendly for non‐R users. Other platforms like ODFM [27] and HoloFood [28] are specialized for limited food types and lack comprehensive microbiome data analysis and visualization modules. It is urgent to establish a comprehensive food microbiome database with user‐friendly data analysis and visualization capabilities to facilitate food science research.

Here, we present Food Microbiome Database (FoodMicroDB), a microbiome database focused on gathering microbiome data across various food categories, and providing data analysis and visualization functionalities. The database features: (a) curated host–food–organism relationship for each project, enabling organized data management by food and organism; (b) a consistent workflow for data analysis and metadata curation, facilitating cross‐data set analysis and comparisons; (c) comprehensive data analysis and visualization modules that effectively present the microbial composition and group comparison results; (d) a unique time‐series analysis module designed to track the microbial changes across time in longitudinal microbiome studies. FoodMicroDB serves as a valuable reference for researchers who are interested in exploring the microbiome compositions of specific foods as well as the distributions and abundance comparisons of microbes across various food hosts.

## 2 RESULTS

### 2.1 Overview of the database

**Figure 1:**
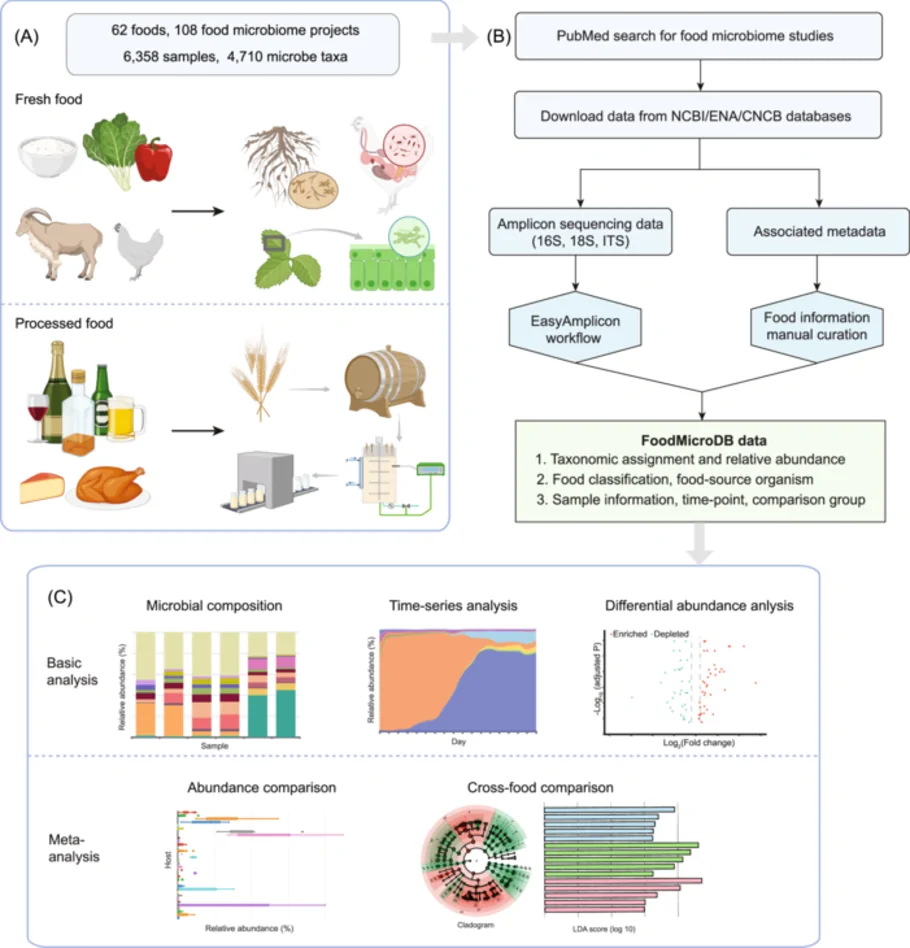
Food Microbiome Database (FoodMicroDB) overview. (A) Schematic of food microbiome projects included in the database, encompassing both fresh foods (agricultural products) and processed foods. (B) Flowchart detailing the data collection and construction of the database. (C) Main functions of FoodMicroDB: Basic analysis, which includes microbial composition, time‐series analysis, and differential abundance analysis; and meta‐analysis, which covers microbial abundance comparison across hosts, and microbial marker identifications by cross‐food analysis.

FoodMicroDB collected 6358 food amplicon data from 108 meta‐taxonomic projects spanning 62 foods. In addition to the project–sample relationships, we organized the collected data by their associated foods and food‐sourced organisms, using the curated host–food–organism relationships for each sample. The foods covered in the database include both fresh foods (i.e., raw agricultural products) and processed foods. For fresh foods, the studies primarily focus on characterizing the microbiomes of various plant and animal components, including plant rhizosphere soil, fruit surface, animal guts, and so forth. In contrast, for processed foods, the research investigates microbial changes during fermentation or production processes (Figure 1A). We categorized the foods by Periodic Table of Food Initiative (PTFI) food classification method [29]. Among the total of 62 foods, 35 are plant food products, 25 belong to animal food products and two others based on their source materials (Figure S1A). In particular, we have 29 fermented foods (47%, Figure S1B), including fermented beverages, dairy products, meat products, and so forth.

To ensure consistency in the analysis method, we downloaded all amplicon sequencing data from public databases (NCBI SRA/ENA/CNCB GSA) and re‐analyzed them using a uniform amplicon sequencing data analysis workflow, EasyAmplicon [30]. The microbial composition data and metadata comprise the original data stored, the FoodMicroDB (Figure 1B). To visualize the microbiome data on a per project basis, we developed microbial composition, time‐series analysis, and differential abundance analysis modules. In addition, the microbial abundances can be examined across hosts under the microbe information page. Microbial markers (significantly changed microbes in relative abundance through group comparisons) for certain foods or food part are identified through the cross‐food analysis module (Figure 1C).

### 2.2 Microbial composition visualization on a per‐project basis

**Figure 2:**
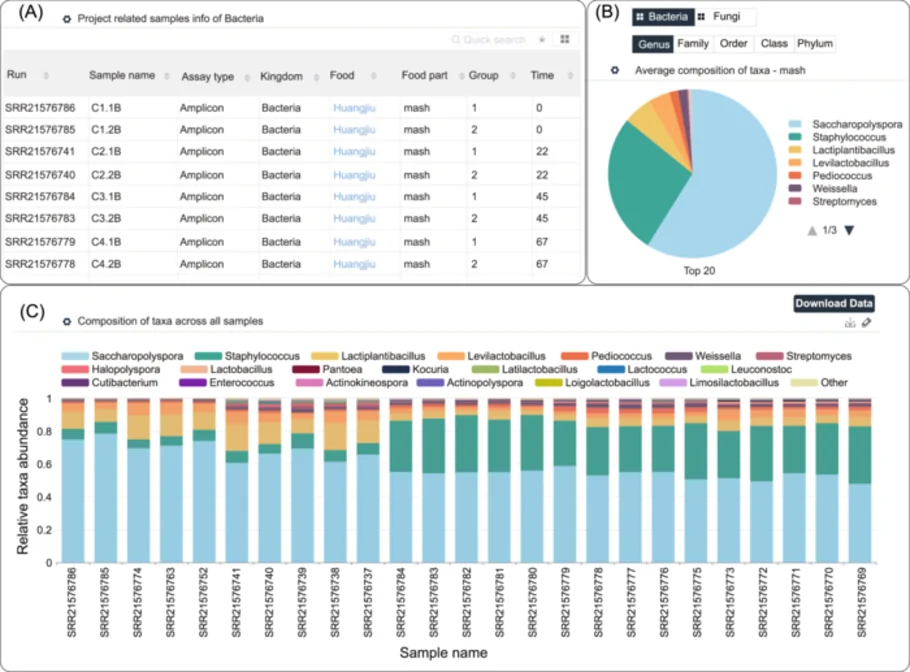
Microbial composition analysis module. (A) Sample information table showing curated sample metadata using Huangjiu fermentation study (PRJNA880416) as an example. “Food,” “Food part,” “Group,” and “Time” information are manually curated from the original publication, while the others are derived from project metadata. (B) The pie chart depicts the average relative abundance of microbe taxa at the genus level. Users can select kingdom (bacteria, archaea, or fungi) and taxonomic levels (from phylum to genus) of interest using the selection tab above. (C) The stacked bar plot shows the relative abundance of the microbes across all samples of the same project. Sample IDs from each run are extracted from the original metadata table of the sequencing data and are listed on the *x*‐axis of the bar plot. For (B) and (C), the legend identifies the top 20 most abundant taxa, with all others categorized as “Other.” The raw data are downloaded by clicking the data table icon at the top right of the plot.

To visualize the microbiome data after the EasyAmplicon analysis workflow, we developed the microbial composition analysis module. For each project, sample information was extracted from raw metadata and then manually curated with host–food relationship which shows as “Food part” and “Food” in each sample entry. Taking the Huangjiu fermentation study (BioProject ID: PRJNA880416) [31] as an example, it studied microbiota profiles of the mash of Huangjiu during the fermentation processes, which is crucial for Huangjiu's flavor. Therefore, “Mash” is the food part in this study (Figure 2A). Additionally, the project has both 16S rRNA and ITS sequencing data, of which the analyzed results were displayed through distinct tabs on the web page (Figure 2B). Taxa relative abundances were organized by food part and displayed in two formats: as averages across samples and individually (Figure 2B,C). In addition to the visualization, the processed data are also downloadable on the web page.

### 2.3 Time‐series and differential abundance analysis modules for projects with curated experimental designs

**Figure 3:**
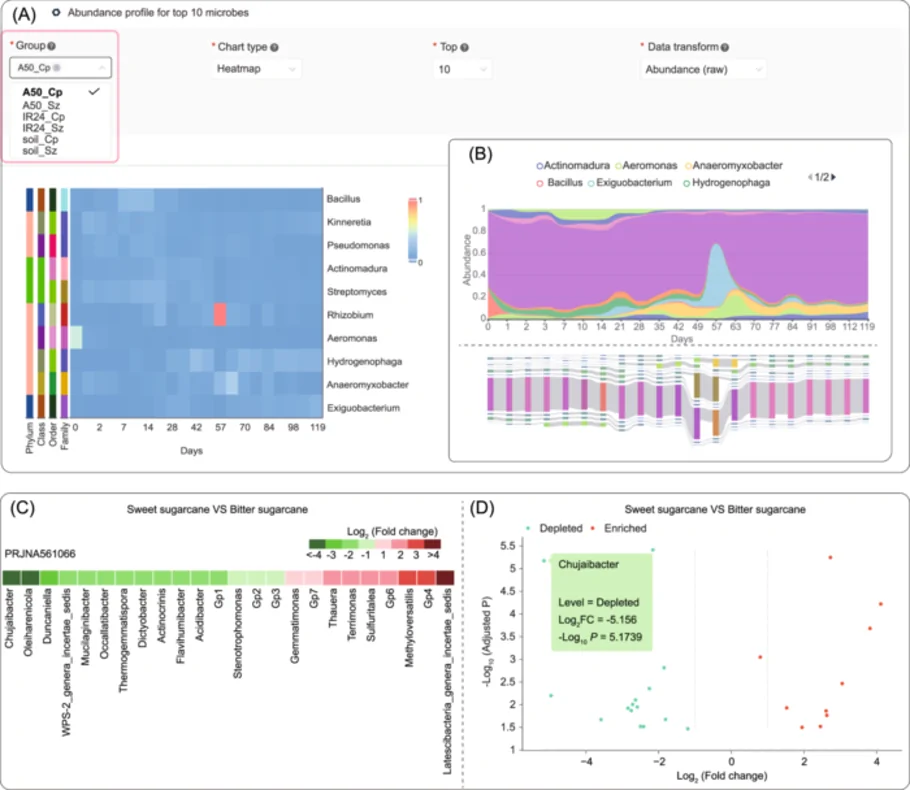
Advanced microbiome data visualization modules. (A, B) Time‐series project data visualization with group selection option, featuring data from the rice root and rhizosphere soil microbiome study (PRJNA435900). The top 10 most abundant microbes across all time points are visualized by heatmap (A) and alluvial plot/Sankey plot (B). The red box highlights the group selection area, where labels such as “A50_Cp,” “A50_Sz” represent different sample groups. Users can select one or multiple groups. (C, D) Differential abundance analysis for projects with two‐group comparison design, using sugarcane rhizosphere soil study (PRJNA561066) as an example. The differentially abundant microbes (at genus level) between sweet and bitter sugarcanes are displayed in a heatmap (C) and a volcano plot (D). Microbes are identified as differentially abundant using the “edgeR” method with *p* value < 0.05, FDR < 0.2, and relative abundance > 0.1%. In (D), details of the markers appear when the mouse hovers over the dot.

To better visualize longitudinal microbiome data, we implemented the time‐series module with more specialized visualization and analysis. We selected the top most abundant microbes based on the summed relative abundance across all samples and displayed their abundances over time by heatmap, alluvial plot, and Sankey plot (Figure 3A,B). The shared microbes among all‐time point groups were displayed by flower plot. For projects that have multiple groups, we provided the multichoice option for users to select groups of interest. As an example, PRJNA435900 is a data set derived from a time‐course study on rice root microbiota [14], where the roots of two rice varieties and bulk soil from two different farms were studied. When selecting “A50_Cp” group in the multiple‐selection box and clicking the submit button, the plot is shown accordingly (Figure 3A,B). It shows that for rice root of the A50_Cp (Nipponbare rice cultivar grown in Changping farm) samples, the top 10 bacteria at the genus level overall are not prominent while the others (the purple in the alluvial plot) are the main composition. Through the group selection box, users can quickly visualize the data from samples of interest.

We also developed differential abundance (DA) analysis module to quickly visualize results for group comparison studies. Projects with two‐group design were selected, and the DA analysis is performed to obtain the significantly changed taxa in relative abundances for the comparison group. The precomputed results were then visualized on the interactive heatmap and volcano plot. We used the sugarcane rhizosphere soil microbiome study [15] as an example, which aims to link the rhizosphere microbiome with the sugarcane flavor. DA analysis was conducted for the sweet versus bitter sugarcanes comparison group, discriminatory microbial taxa at the genus level were identified (Figure 3C,D). Users could hover over each point to check the detailed information of DA species. Interestingly, our results, which show the enrichment of *Chujaibacter* and *Stenotrophomonas* in sweet sugarcanes, match the two genera listed in the top 20 taxa from the original publication, thereby validating the reliability of our analysis.

### 2.4 Examine microbial abundance across food‐associated hosts

**Figure 4:**
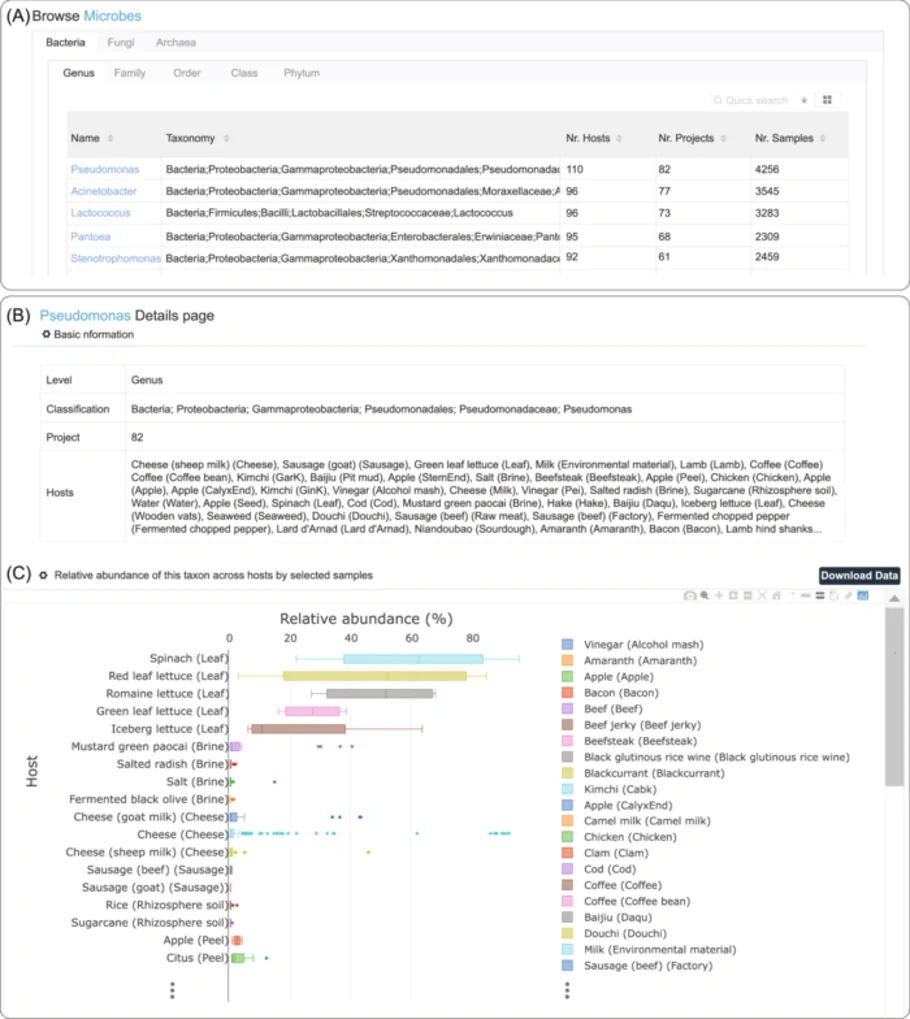
Distribution and abundance of microbes across food hosts. (A) Overview of the “Bacteria Microbes” page. Users can switch kingdoms (bacteria, fungi, and archaea) and taxonomic levels (from phylum to genus) using the tab above. (B) Microbe detail page showing taxonomic classification and host information of *Pseudomonas*. (C) Distribution and abundance of *Pseudomonas* in various food‐related hosts shown by a box plot.

The FoodMicroDB database harbors a total of 4710 food‐associated microbes extracted from the deposited datasets, including 3182 bacteria, 123 archaea, and 1405 fungi (Figure 4A). The details of the microbes were shown on the detail page, with microbe's host information. The relative abundance across all hosts was shown as a boxplot. Users can select microbes of interest using the search box to examine their distribution and relative abundances across hosts. As an example, *Pseudomonas* is the most prevalent bacteria genus and appear in 82 projects and 110 hosts. It is an abundant taxon in food hosts such as spinach, red leaf lettuce, and Romaine lettuce leaves, comprising on average more than 50% of the total composition (Figure 4B,C). Food‐pathogenic microbes may be of general interest, and we examined the abundances of the two bacterial genera including *Salmonella* and *Listeria*, and found they have only 37 and 13 hosts, respectively. Notably, *Salmonella* only appears to be nontrivial and occupied over 5% in a Kimchi samples (Gink samples, or ginger‐derived kimchi). While Listeria is on average lowly abundant as less than 0.5% in all hosts, but shows to have large variations in Lard d'Arnad samples (Figure S3).

### 2.5 Identify microbial markers of distinct foods with cross‐food analysis

**Figure 5:**
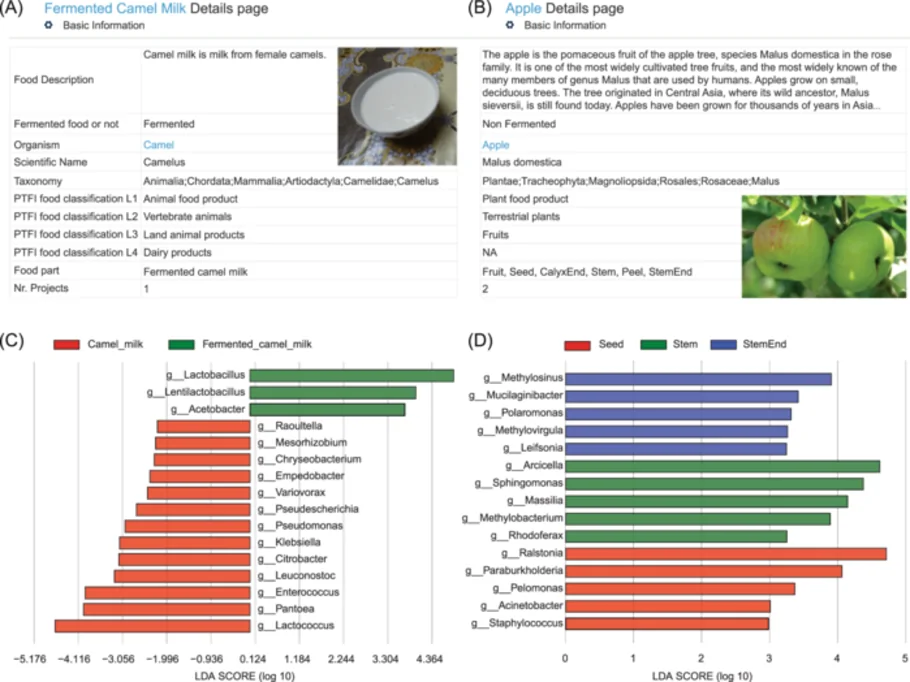
Cross‐food analysis module. (A, B) Food pages for “Fermented camel milk” and “Apple,” respectively. These pages display food metadata including the organism and taxonomy, food classification by Periodic Table of Food Initiative (PTFI) method, food part, and associated projects. The image for fermented camel milk is sourced from Wikipedia (https://en.wikipedia.org/wiki/Chal), and the apple image from FooDB (https://www.foodb.ca/foods/FOOD00105). (C, D) Differential bacterial markers were identified by the cross‐food analysis module using LEfSe. In (C), “fermented camel milk” and “camel milk” are compared, with only genus taxa to display. In (D), comparisons are made between the seed, stem, and stem end of the apple, with only the top 5 markers at the genus level displayed for clarity.

To find microbial markers for certain foods or food parts, we developed an interactive cross‐food analysis module. All food or their different parts deposited in the databases can be selected for such analysis. Using data from the camel milk microbiome characterization study (with food information displayed in Figure 5A) [31], we found the unique bacteria in fermented and fresh camel milk with LEfSe analysis (Figure 5C). We found *Lactococcus* and *Acetobacter* as the enriched genus in the fermented camel milk group, while *Lactobacillus* and *Leuconostoc* in the fresh camel milk and a few more taxa in each group, which are largely consistent with the original findings. The same comparison was conducted for the ITS data (Figure S2) and the most significantly changed genus for the camel milk group is *Kazachstania*, which is consistent with the results from the original publication, further validating our analysis.

Moreover, comparison across different parts of foods is supported. As an example, for data from the apple microbiome study [32] (with the food information shown in Figure 5B), we performed a multigroup analysis on three distinct parts of the apple, that is, “stem,” “stem end,” and “seed.” The resulting microbial markers for each part are shown in the bar plot in Figure 5D. This comparison was not performed in the original study, highlighting how our tool can enhance data exploration beyond the initial research.

Additionally, the website has an advanced function for searching microbes in time‐series projects. Under the “Cross‐study”—“Time‐series projects search” section, users can also search the microbes and find all projects containing those microbes as their top abundant microbes. Alternatively, users can provide the sequences instead of the microbe names.

## 3 DISCUSSION

FoodMicroDB is a platform specialized for archiving microbiome data from food biome studies and providing versatile data analysis and visualization tools. It aims to serve the food microbiology research field by facilitating the microbiome data accessibility and reusability. Managing diverse studies related to the complex system of food is challenging. To address this, each sample is assigned a host–food–organism relationship, allowing data to be systematically organized by associated project and food/organism. This approach ensures that microbiome studies on any edible substance and their related components are inclusively cataloged in our database. We use a food classification method derived from PTFI to categorize food products into specific groups for easier inventory management, which labeling food based on their main source materials. The platform currently contains 108 food microbiome projects and 6358 amplicon data (16S/18S/ITS). We re‐analyzed all data using unified workflow, reducing batch effect from analysis methods, and facilitate cross‐study analysis. Importantly, FoodMicroDB offers unique and versatile microbiome data analysis and visualization modules. These tools enable users to quickly access microbiome data related to their specific food or microbe of interest. This feature is distinctive and not yet available in other food microbiome databases, such as FoodMicroBioNet, HoloFood, and ODFM [26, 27, 28].

Despite its strengths, the current version of FoodMicroDB has some limitations. It contains only amplicon sequencing data, indicating a potential for expansion to include more types of data (such as metagenomics and metatranscriptomics data) in the future. So far, the number of amplicon projects in the databases is not the largest database regarding food amplicon data, which is smaller than FoodMicroBioNet v5, showing the potential for including more data in the future. Additionally, there is room for the development of more analysis functions to enhance its utility further. There is a growing trend of using online omics analysis platforms like Majorbio Cloud (commercial), Wekemo Bioincloud, and ImageGP [33, 34, 35], which enable users to upload their own data for various analyses. We will incorporate this feature, allowing users to upload their own data for visualizations and to perform cross‐study comparisons with data from our database. Lastly, we plan to utilize the extensive sample metadata we have meticulously curated, including details like food part, group, and time, to develop more interactive analysis modules. This encompasses online differential expression analysis, alpha diversity among different groups, and highlighting microbial markers especially for the potential health benefits from food intake. These enhancements can be tackled in the subsequent development phases to guarantee that the platform can fulfill the escalating demands of the research community.

## 4 CONCLUSION

In this study, we introduced FoodMicroDB, an online database specialized for food microbiome research. It archived 6358 amplicon data from 108 meta‐taxonomic projects covering 62 foods and 4710 taxa of bacteria, archaea, and fungi. FoodMicroDB is the first food microbiome database that uniquely providing microbiome data analysis and visualization functionalities, including composition, time‐series analysis, differential expression analysis. It also features consistent data analysis enabling cross‐host data integration and comparisons. The delicate food data curation, such as host–food–organism relationship for each project, allowing for easy search and navigation. This freely accessible online‐browsable database will become an important and valuable resource platform for the food microbiome research field, benefiting researchers in the food, as well as for general plant and animal microbiome area. In the future, we will keep updating FoodMicroDB with more data, and develop more utilities to better serve the community.

## 5 METHODS

### 5.1 Study collection

To collect food microbiome studies, we searched the PubMed database (https://pubmed.ncbi.nlm.nih.gov/) using the keywords “[Food]” AND “microbiome” through August 2023, where the “[Food]” was replaced with the name of a specific food name, such as “apple,” “cheese,” “chicken,” “fish,” and so forth. We only kept studies with accessible raw sequencing data.

### 5.2 Data curation and data analysis

Using the data accession numbers, we downloaded the raw sequencing data and metadata from public databases. For data housed in the NCBI SRA (https://www.ncbi.nlm.nih.gov/sra) and ENA (https://www.ebi.ac.uk/ena), we used the command‐line tools from SRA toolkit. For data from the CNCB GSA (https://ngdc.cncb.ac.cn/gsa/), ftp was used for downloading.

We extracted sample metadata from the original uploaded metadata and publication, and curated a few extra metadata including “Group,” “Time,” “Host,” “Food,” “Food part,” and “Organism.” The missing latitude and longitude of the study samples were found by searching online using the city or country names provided in the publication. The organism taxonomy, food description, and food pictures were collected through the original publications, Wikipedia (https://www.wikipedia.org/), and FooDB website (https://foodb.ca/). The food classification is based on the PTFI method [29]. The taxonomy of microbes was curated based on the RDP (16S v18) [36] and UNITE (2021‐05‐10 all eukaryotes) [37] databases using in‐house scripts.

We applied EasyAmplicon workflow to reanalyze the amplicon sequencing data to obtain taxonomic assignment and relative abundance. First, if the original sequences produced by the sequencing platform are paired‐end sequences, they need to be merged to acquire amplified fragment sequences; however, single‐end sequences do not require this step. Subsequently, the barcodes and primers are eliminated from the original read sequences, followed by quality control and filtering out low‐quality amplified fragment sequences with a sequencing error rate not exceeding 0.01. The primer length is determined based on the description in the publication and manual inspection of the original sequence. All these steps can be accomplished using VSEARCH [38]. Then, the representative sequences are selected through sequence deduplication, noise elimination, and chimera removal using USEARCH [39], and the frequency of representative sequences in each sample is quantified to construct a feature table. Finally, the representative sequences are classified at the levels of kingdom, order, family, genus, species, and subspecies using VSEARCH and USEARCH, respectively, for bacteria/archaea and fungi, with the RDP and UNITE databases utilized for taxonomic annotation [40].

### 5.3 Statistical methods

In the differential abundance (DA) analysis, scripts from the EasyAmplicon workflow were utilized with default settings. Specifically, the edgeR [41] method was selected within the “compare” function, which applies a negative binomial generalized log‐linear model to the amplicon read‐counts matrix. Differential abundance taxa were identified using the criteria of a relative abundance greater than 0.1%, and a significance threshold set at a *p* value of less than 0.05 and FDR less than 0.2. In the cross‐food analysis module, Linear discriminant analysis Effect Size (LEfSe) [42] analysis was conducted using the encapsulated script in ImageGP [35] with default parameters. A linear discriminant analysis (LDA) score of 2 was set as the cutoff for identifying marker taxa. Markers from the Phylum to Genus levels were all identified.

### 5.4 Database construction and web implementation

The open‐source data management system Mysql (https://www.mysql.com/) is used for curated metadata saving and accessing. Abundance profiles of taxa were stored in hdf5 format for fast access and calculation. The website is implemented as a web application using Javascript and HTML for front‐end development. The used core JavaScript libraries include Vue.js (https://vuejs.org/) as the main frontend framework, plotly.js (https://plotly.com/), D3.js (https://d3js.org/) and Echarts.js (https://echarts.apache.org/zh/index.html) for interactive visualizations and data explorations. High‐level web framework Django (https://www.djangoproject.com/) is used for backend data preprocess and data analysis. The global search function is based on the Elasticsearch module (https://www.elastic.co/elasticsearch/).

## AUTHOR CONTRIBUTIONS

**Yahui Li**: Investigation; methodology; data curation; data analysis; writing—original draft; writing—review and editing; visualization. **Hujie Lyu**: Data curation; data analysis; visualization. **Haifei Yang**: Data curation; data analysis. **Zhichen Ju**: Investigation; data curation; data analysis. **Chuang Ma**: Data analysis; data curation. **Huiyu Hou**: Writing—review and editing. **Yao Wang**: Writing—review and editing. **Yuanping Zhou**: Writing—review and editing. **Yunyun Gao**: Writing—review and editing. **Junbo Yang**: Writing—review and editing. **Shanshan Xu**: Website testing and suggestions. **Defeng Bai**: Website testing and suggestions. **Hao Luo**: Website testing and suggestions. **Salsabeel Yousuf**: Website testing and suggestions. **Tianyuan Zhang**: Website testing and suggestions. **Jiani Xun**: Website testing and suggestions. **Meiyin Zeng**: Website testing and suggestions. **Heyuan Qi**: Methodology. **Tong Chen**: Conceptualization; methodology; writing—review and editing. **Yong‐Xin Liu**: Conceptualization; methodology; funding acquisition; project administration; writing—review and editing; supervision.

## CONFLICT OF INTEREST STATEMENT

The authors declare no conflict of interest.

## ETHICS STATEMENT

No animals or humans were involved in this study.

## Supporting information


**Figure S1.** Statistics of foods included in FoodMicroDB.
**Figure S2.** Differential fungal markers between “Fermented camel milk” and “Camel milk” were identified by the cross‐food analysis.
**Figure S3.** Distribution and abundance of two typical pathogenic bacterial genera.

## Data Availability

The data that support the findings of this study are openly available in Github repository for FoodMicroDB at https://github.com/yli085/FoodMicroDB. FoodMicroDB is freely available at https://www.bic.ac.cn/FoodMicro/. The project metadata and analysis codes are publicly shown at https://github.com/yli085/FoodMicroDB. Supplementary materials (figures, scripts, graphical abstract, slides, videos, Chinese translated version and update materials) may be found in the online DOI or iMeta Science http://www.imeta.science/imetaomics/.
