# Supplementary material for: FoodMicroDB: A microbiome database for composition and time‐series research in food
Source: IMetaOmics. 2024 Nov 1;1(2):e40. doi: 10.1002/imo2.40 (PMC12806411; doi:10.1002/imo2.40)
Supplement: Supplementary file 1 — Figure S1. Statistics of foods included in FoodMicroDB. Figure S2. Differential fungal markers between “Fermented camel milk” and “Camel milk” were identified by the cross‐food analysis. Figure S3. Distribution and abundance of two typical pathogenic bacterial genera. [file IMO2-1-e40-s001.docx]

Supporting information to:

**FoodMicroDB: a microbiome database for composition and time-series research in food**

**Running title**: FoodMicroDB: a database for food microbiome research

Yahui Li^1#^, Hujie Lyu^2#^, Haifei Yang^1, 3#^, Zhicheng Ju^4^, Chuang Ma^5^, Huiyu Hou^1^, Yao Wang^1^, Yuanping Zhou^1,6^, Yunyun Gao^1^, Junbo Yang^1^, Shanshan Xu^7^, Defeng Bai^1^, Hao Luo^1^, Salsabeel Yousuf^1^, Tianyuan Zhang^1^, Jiani Xun^1^, Meiyin Zeng^1^, Heyuan Qi^8^, Tong Chen^9^*, Yong-Xin Liu^1^*

^1^Agricultural Genomics Institute at Shenzhen, Chinese Academy of Agricultural Sciences, Shenzhen 518120, China

^2^Department of Life Sciences, Imperial College of London, London SW7 2AZ, United Kingdom

^3^College of Life Sciences, Qingdao Agricultural University, Qingdao 266000, China

^4^Department of Ocean Science, The Hong Kong University of Science and Technology, Hong Kong SAR, 999077, China

^5^Anhui Agricultural University, Hefei 230036, China

^6^Zhanjiang Key Laboratory of Human Microecology and Clinical Translation Research, the Marine Biomedical Research Institute, College of Basic Medicine, Guangdong Medical University, Zhanjiang, Guangdong, 524023, China

^7^School of Food and Biological Engineering, Hefei University of Technology, Hefei 230009, China

^8^Institute of Microbiology, Chinese Academy of Sciences, Beijing, 100101, China

^9^State Key Laboratory for Quality Ensurance and Sustainable Use of Dao-di Herbs, National Resource Center for Chinese Materia Medica, China Academy of Chinese Medical Science, Beijing 100000, China

^#^ These authors contributed equally: Yahui Li, Hujie Lyu, and Haifei Yang

* Correspondence: chent@nrc.ac.cn (Tong Chen), [liuyongxin@caas.cn](mailto:liuyongxin@caas.cn) (Yong-Xin Liu)

**Supplementary figures**

**
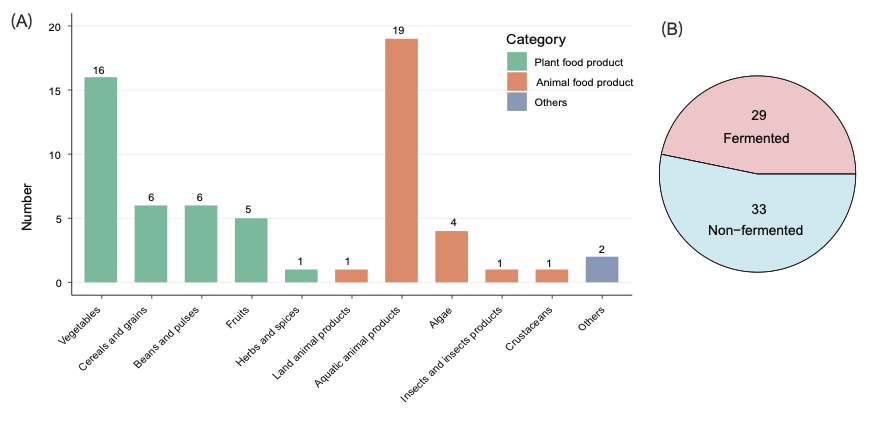
**

**Figure S1. Statistics of foods included in FoodMicroDB.** (A) Food category statistics using classification method from the Periodic Table of Food Initiative (PTFI). (B) Numbers of fermented foods and non-fermented foods.


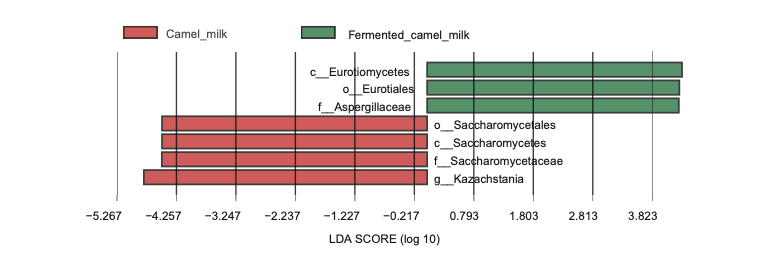


**Figure S2. Differential fungal markers between “Fermented camel milk” and “Camel milk” were identified by the cross-food analysis.**

**
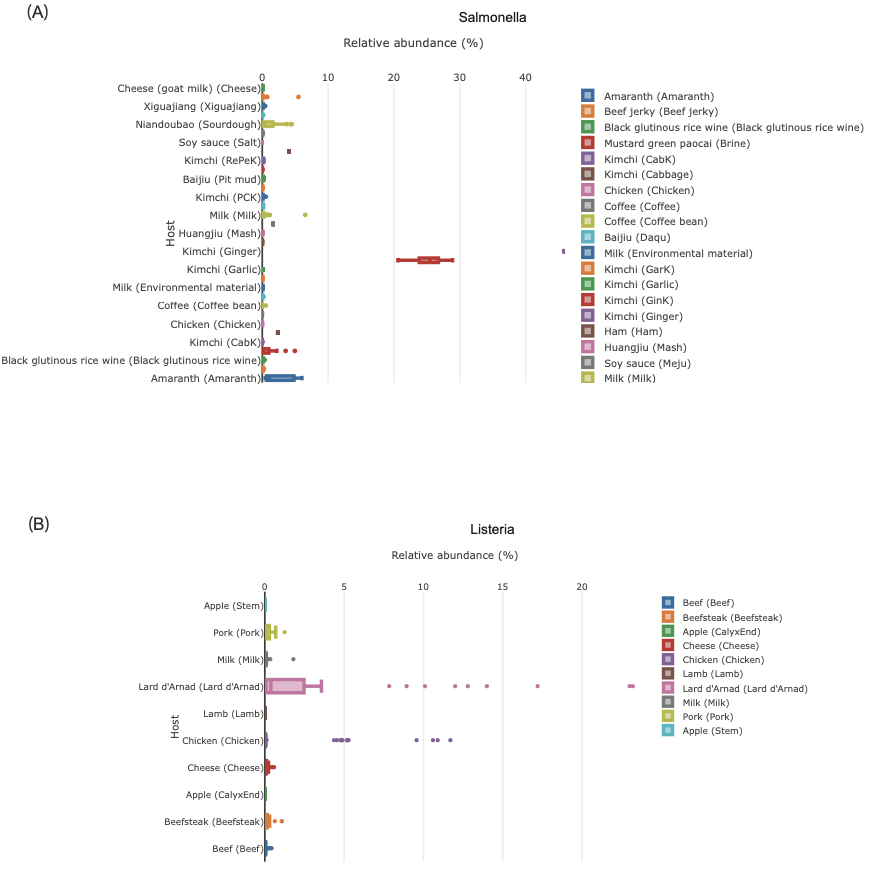
**

**Figure S3. Distribution and abundance of two typical pathogenic bacterial genera.** (A) *Salmonella*. (B) *Listeria*.
